# Clustered Environments and Randomized Genes: A Fundamental Distinction between Conventional and Genetic Epidemiology 

**DOI:** 10.1371/journal.pmed.0040352

**Published:** 2007-12-11

**Authors:** George Davey Smith, Debbie A Lawlor, Roger Harbord, Nic Timpson, Ian Day, Shah Ebrahim

**Affiliations:** 1 Department of Social Medicine, University of Bristol, Bristol, United Kingdom; 2 Medical Research Council Centre for Causal Analyses in Translational Epidemiology, University of Bristol, Bristol, United Kingdom; 3 Department of Epidemiology and Population Health, London School of Hygiene and Tropical Medicine, London, United Kingdom; University of Oxford, United Kingdom

## Abstract

**Background:**

In conventional epidemiology confounding of the exposure of interest with lifestyle or socioeconomic factors, and reverse causation whereby disease status influences exposure rather than vice versa, may invalidate causal interpretations of observed associations. Conversely, genetic variants should not be related to the confounding factors that distort associations in conventional observational epidemiological studies. Furthermore, disease onset will not influence genotype. Therefore, it has been suggested that genetic variants that are known to be associated with a modifiable (nongenetic) risk factor can be used to help determine the causal effect of this modifiable risk factor on disease outcomes. This approach, mendelian randomization, is increasingly being applied within epidemiological studies. However, there is debate about the underlying premise that associations between genotypes and disease outcomes are not confounded by other risk factors. We examined the extent to which genetic variants, on the one hand, and nongenetic environmental exposures or phenotypic characteristics on the other, tend to be associated with each other, to assess the degree of confounding that would exist in conventional epidemiological studies compared with mendelian randomization studies.

**Methods and Findings:**

We estimated pairwise correlations between nongenetic baseline variables and genetic variables in a cross-sectional study comparing the number of correlations that were statistically significant at the 5%, 1%, and 0.01% level (α = 0.05, 0.01, and 0.0001, respectively) with the number expected by chance if all variables were in fact uncorrelated, using a two-sided binomial exact test. We demonstrate that behavioural, socioeconomic, and physiological factors are strongly interrelated, with 45% of all possible pairwise associations between 96 nongenetic characteristics (*n* = 4,560 correlations) being significant at the *p* < 0.01 level (the ratio of observed to expected significant associations was 45; *p*-value for difference between observed and expected < 0.000001). Similar findings were observed for other levels of significance. In contrast, genetic variants showed no greater association with each other, or with the 96 behavioural, socioeconomic, and physiological factors, than would be expected by chance.

**Conclusions:**

These data illustrate why observational studies have produced misleading claims regarding potentially causal factors for disease. The findings demonstrate the potential power of a methodology that utilizes genetic variants as indicators of exposure level when studying environmentally modifiable risk factors.

## Abstract 2

In a cross-sectional study Davey Smith and colleagues show why observational studies can produce misleading claims regarding potential causal factors for disease, and illustrate the use of mendelian randomization to study environmentally modifiable risk factors.

## Editors' Summary

**Background.:**

Epidemiology is the study of the distribution and causes of human disease. Observational epidemiological studies investigate whether particular modifiable factors (for example, smoking or eating healthily) are associated with the risk of a particular disease. The link between smoking and lung cancer was discovered in this way. Once the modifiable factors associated with a disease are established as causal factors, individuals can reduce their risk of developing that disease by avoiding causative factors or by increasing their exposure to protective factors. Unfortunately, modifiable factors that are associated with risk of a disease in observational studies sometimes turn out not to cause or prevent disease. For example, higher intake of vitamins C and E apparently protected people against heart problems in observational studies, but taking these vitamins did not show any protection against heart disease in randomized controlled trials (studies in which identical groups of patients are randomly assigned various interventions and then their health monitored). One explanation for this type of discrepancy is known as confounding—the distortion of the effect of one factor by the presence of another that is associated both with the exposure under study and with the disease outcome. So in this example, people who took vitamin supplements might have also have exercised more than people who did not take supplements and it could have been the exercise rather than the supplements that was protective against heart disease.

**Why Was This Study Done?:**

It isn't always possible to check the results of observational studies in randomized controlled trials so epidemiologists have developed other ways to minimize confounding. One approach is known as mendelian randomization. Several gene variants have been identified that affect risk factors. For example, variants in a gene called APOE affect the level of cholesterol in an individual's blood, a risk factor for heart disease. People inherit gene variants randomly from their parents to build up their own unique genotype (total genetic makeup). Consequently, a study that examines the associations between a gene variant and a disease can indicate whether the risk factor affected by that gene variant causes the disease. There should be no confounding in this type of study, the argument goes, because different genetic variants should not be associated with each other or with nongenetic variables that typically confound directly assessed associations between risk factors and disease. But is this true? In this study, the researchers have tested whether nongenetic risk factors are confounded by each other and also whether genetic variants are confounded by nongenetic risk factors and also by other genetic variants

**What Did the Researchers Do and Find?:**

Using data collected in the British Women's Heart and Health Study, the researchers calculated how many pairs of nongenetic variables (for example, frequency of eating meat, alcohol intake) were significantly correlated with each other. That is, the number of pairs of nongenetic variables in which a high correlation between both variables occurred in more study participants than expected by chance. They compared this number with the number of correlations that would occur by chance if all the variables were totally independent. When the researchers assumed that 1 in 100 combinations of pairs of variables would have been correlated by chance, the ratio of observed to expected significant correlations was seen 45 times more frequently than would be expected by chance. When the researchers repeated this exercise with genetic variants, the ratio of observed to expected significant correlations was 1.58, a figure not significantly different from 1. Similarly, the ratio of observed to expected significant correlations when pairwise combinations between genetic and nongenetic variants were considered was 1.22.

**What Do These Findings Mean?:**

These findings have two main implications. First, the large excess of observed over expected associations among the nongenetic variables indicates that many nongenetic modifiable factors occur in clusters—for example, people with healthy diets often have other healthy habits. Researchers doing observational studies always try to adjust for confounding but this result suggests that this adjustment will be hard to do, in part because it will not always be clear which factors are confounders. Second, the lack of a large excess of observed over expected associations among the genetic variables (and also among genetic variables paired with nongenetic variables) indicates that little confounding is likely to occur in studies that use mendelian randomization. In other words, this approach is a valid way to identify which environmentally modifiable risk factors cause human disease.

**Additional Information.:**

Please access these Web sites via the online version of this summary at http://dx.doi.org/10.1371/journal.pmed.0040352.

- Wikipedia has pages on epidemiology and on mendelian randomization (note: Wikipedia is a free online encyclopedia that anyone can edit; available in several languages).
- Epidemiology for the Uninitiated is a primer from the *British Medical Journal*
- Information is available on the British Women's Heart and Health Study

## Introduction

Observational epidemiology has had notable successes, but also high-profile failures, in that it has identified many modifiable exposures apparently increasing or decreasing disease risk that have been revealed by randomized controlled trials to be noncausal [1]. The explanation in many of these cases is likely to be that confounding—by lifestyle and socioeconomic factors, or by baseline health status and treatment effects—is responsible for observed associations [2,3]. Many potentially health-modifying factors (such as use of antioxidant vitamin supplements) will be strongly related to such confounding factors [4]. Other factors that can lead to observational associations being poor predictors of causal effects include reverse causation (in which early stages of the disease process influence the exposure, rather than vice versa), imprecision in effect estimates (due to inadequate sample size), information and selection biases, and distortion of the available scientific literature that may be introduced by the processes of publication of research [5–9].

One approach to such problems in observational epidemiology is mendelian randomization [10]. The basic principle utilised in such studies is that if a genetic variant influences an environmentally modifiable risk factor that itself alters disease risk, then the genetic variant should be associated with disease risk. Further, the causal effect of the environmentally modifiable risk factor on disease risk can be calculated (under certain assumptions [11]) from the magnitude of the genetic variant's associations with disease risk and with the environmentally modifiable risk factor. The advantage here is that the genetic variant should not be associated with the confounding lifestyle, socioeconomic, or medical care factors that distort the study of directly measured exposures and disease [10]. Furthermore, the genetic variant will not be influenced by the early stages of the disease process, and the estimate of the causal effect of interest will thus be immune to the reverse causation that can distort conventionally studied associations [5]. Observational studies of genetic variants may, therefore, have similar properties to intention-to-treat analyses aimed at determining the causal nature of a particular treatment in randomized controlled trials.

Such mendelian randomization studies have been conducted within the cardiovascular and cancer fields. This approach has provided evidence that alcohol intake increases the risk of esophageal cancer [12], and that fibrinogen and C-reactive protein appear not to increase cardiovascular disease risk or adversely influence components of the metabolic syndrome, and are therefore not suitable targets for specific pharmocotherapeutic modification [13–16]. The development of the mendelian randomization concept and the associated terminology has been discussed in detail elsewhere [17].

Despite theoretical reasons why genetic variants should be largely unrelated to many exposures or phenotypic characteristics, it has been suggested that genetic association studies in general, and mendelian randomization approaches in particular [18], are susceptible to confounding. It is suggested that confounding may occur because of the pleiotropic effect of genes (i.e., one variant affecting several phenotypes), linkage disequilibrium between the variant under study and variants influencing other phenotypes, and population substructure [10]. Pleiotropic effects would only confound associations of genotype with disease outcome if any additional pleiotropic effects of the gene were also associated with the disease outcomes of interest. Similarly, if there is linkage disequilibrium between the genotype being used as an instrument and a polymorphism that is associated with the outcome, then confounding of the gene–outcome association may occur, but if the linkage disequilibrium is with a variant that is unrelated to the outcome of interest, this will not confound the association

Population substructure would result in confounding of the genotype–outcome association if subgroups exist within a population that have different genetic histories and different disease risks (for genetic or other reasons) as this would generate misleading associations between genetic variants and phenotypes (so-called “population stratification”). The importance of population stratification has generated a vigorous debate, but it appears that if basic precautions are applied with respect to the ethnicity and population of origin of study sample members, and appropriate analytical strategies are applied, then bias should generally be small [19–24].

Thus, we suggest that conventional observational epidemiology is particularly prone to confounding because nongenetic characteristics are highly associated with each other, perhaps even more so than is generally acknowledged. At the same time, mendelian randomization studies can exploit the general lack of associations between one genetic variant and other genetic variants, and between genetic and nongenetic variables, to provide an unconfounded estimate of the association between factors that the genetic variant directly influences and disease outcomes.[10] As discussed above, there is concern that mendelian randomization studies may, however, be confounded through pleiotropy, linkage disequilibrium, or population stratification. In truth, within the bounds of conventional epidemiological cohorts, no formal exercise has examined the extent to which genetic variants on the one hand, and nongenetic environmental exposures or phenotypic characteristics on the other, tend to be associated with each other—i.e., the degree of confounding that would exist in conventional epidemiological studies compared with mendelian randomization studies. We have therefore examined this issue empirically in the British Women's Heart and Health Study [25].

## Methods

Data from the British Women's Heart and Health Study were used. Full details of the selection of participants and measurements, including DNA extraction and genotyping, have been previously reported [25]. Briefly, women aged 60–79 y were randomly selected from general practitioner lists in 23 British towns. A total of 4,286 women participated, and baseline data (self-completed questionnaire, research nurse interview, physical examination, and primary care medical record review) were collected between April 1999 and March 2001.

We estimated pairwise correlations between 96 nongenetic baseline variables in the study that were continuous, binary, or ordered categorical (see Text S1 for full list of these and details of whether they were continuous or how they were categorised). We compared the number of pairwise correlations that were observed to be statistically significant at the 5%, 1%, and 0.01% level (α = 0.05, 0.01, and 0.0001, respectively) with the number expected by chance if all variables were in fact uncorrelated, using a two-sided binomial exact test. We chose to compare observed to expected significant associations at these values of statistical significance because α = 0.05 and α = 0.01 are the most commonly used values in observational epidemiological studies to indicate departure from the null hypothesis, whereas for genetic associations with complex traits. much smaller *p*-values (of the order of 0.0001 or below) are recommended to avoid false-positive claims. By using three different values of statistical significance, we were able to determine the extent to which our results were driven by the level of significance chosen.

Three of the authors (GDS, DAL, and SE) decided a priori whether they considered that any of the nongenetic variables were measuring the same underlying characteristic or phenotype (e.g., systolic and diastolic blood pressure) or whether there were subgroups that were constituents of an overall variable (e.g., lipid subfractions and total cholesterol). Text S1 provides full details of these groups of phenotypes. In a series of sensitivity analyses, we replaced the variables used in the main analyses with other variables that we had considered to be measuring the same phenotype (e.g., replacing systolic with diastolic blood pressure; see Table S1) and replaced subgroups of variables with their overall variable (e.g., lipid subfractions by total cholesterol; see Table S2). In these sensitivity analyses, the proportion of observed statistically significant correlations (at any of α = 5%, 1%, or 0.01%) were essentially the same as for the main results presented here. For associations between nongenetic variables, we controlled for the effect of age (because many variables will show age-related variation) by calculating age-adjusted coefficients. For the results presented here, age was treated as a continuous variable in standardised regression models. We then repeated all analyses with age entered as dummy variables (i.e., a four-category variable: 60–64, 65–69, 70–74, and 75–79 y), which does not assume that age is linearly associated with the variables. The results from these models did not differ from the models with age entered as a continuous variable.

In order to explore the extent to which the use of genetic variants as indicators of exposure levels is valid for measuring the unconfounded associations of a nongenetic risk factor with outcomes, we examined the correlations between each of 23 genetic variants and each of the 96 nongenetic characteristics. In these analyses, we also compared observed with expected statistically significant correlations at α = 5%, 1%, and 0.01% using a two-sided binomial exact test. Results presented for these associations were not age adjusted since genetic variants should not be associated with age (and in formal tests were not; furthermore, age adjustment did not alter any results). When multiple single nucleotide polymorphisms (SNPs) were deliberately genotyped to form common haplotypes, based on existing literature about such haplotypes, only one of these SNPs (selected at random) was included in the analyses (See Table S3 for details). In a series of sensitivity analyses, we replaced the SNP selected at random with one of the other SNPs in the same haplotype block. The results from these sensitivity analyses did not differ substantively from those presented here.

Variables that were markedly positively skewed were log-transformed and categorical variables were treated as scores (see Table S1 and Text S1). All genetic variants were biallelic polymorphisms and were treated as scores from zero to two, with zero representing homozygotes for the dominant allele, one, heterozygotes, and two, homozygotes for the minor allele (see Table S3).

## Results

### Associations of Nongenetic Characteristics with Each Other

The 96 nongenetic variables generated 4,560 pairwise comparisons, of which, assuming no associations existed, five in 100 (total 228) would be expected to be associated by chance at the 5% significance level (α = 0.05). However, 2,447 (54%) of the correlations were significant at the α = 0.05 level, giving an observed to expected (O:E) ratio of 11, *p* for difference O:E < 0.000001 (Table 1). At the 1% significance level, 45.6 of the correlations would be expected to be associated by chance, but we found that 2,036 (45%) of the pairwise associations were statistically significant at α = 0.01, giving an O:E ratio of 45, *p* for difference O:E < 0.000001 (Table 2). At the 0.01% significance level, 0.456 of the correlations would be expected to be associated by chance, but we found that 1,378 (30%) were significantly associated at α = 0.0001, giving an O:E ratio of 3,022, *p* for difference O:E < 0.000001.

**Table 1:**
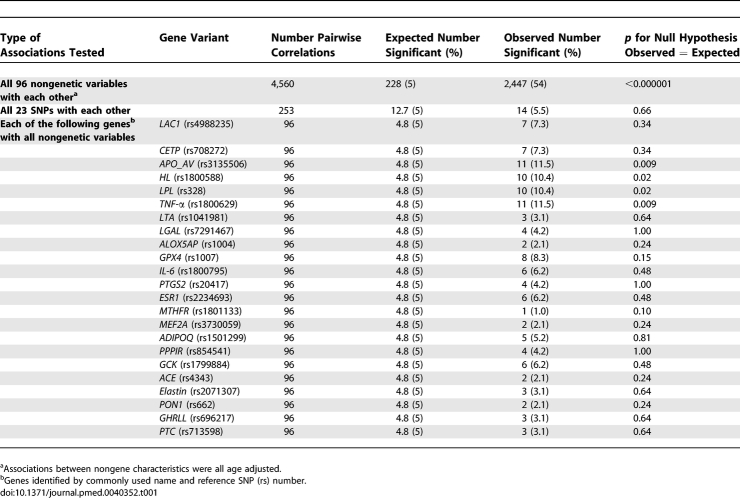
Comparison of Observed to Expected Number of Statistically Significant (at *p* = 0.05) Correlations

**Table 2:**
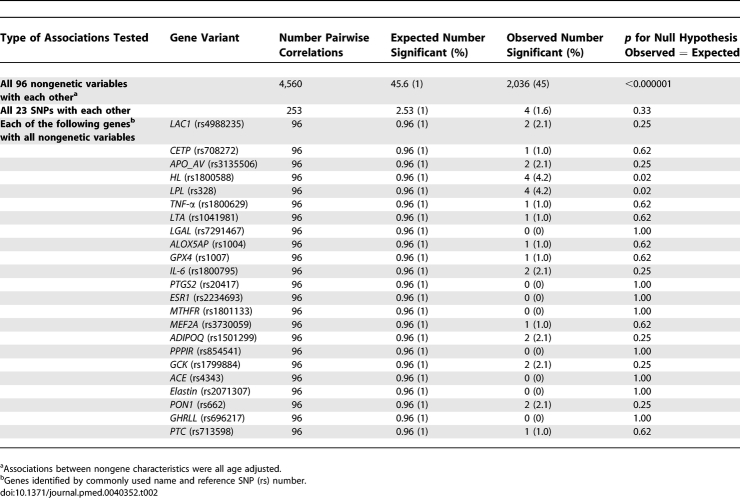
Comparison of Observed to Expected Number of Statistically Significant (at *p* = 0.01) Correlations

**Figure 1:**
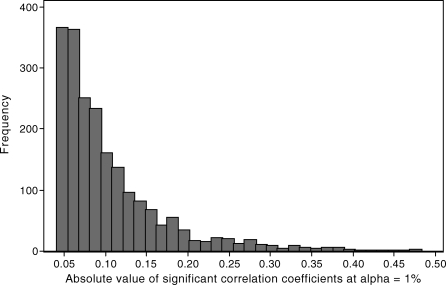
Histogram of Statistically Significant (at α = 1%) Age-Adjusted Pairwise Correlation Coefficients between 96 Nongenetic Characteristics. British Women Aged 60–79 y

Figure 1 shows the histogram of magnitudes of age-adjusted partial correlation coefficients that were significant at the *p* < 0.01 level. At both α = 0.05 and α = 0.01, the median magnitude of the statistically significant age-adjusted partial correlation coefficients was 0.08 (interquartile range, 0.06 to 0.13). At α = 0.0001, the median magnitude of the statistically significant, age-adjusted partial correlation coefficients was 0.11 (interquartile range, 0.09 to 0.16).

### Associations of Genetic Characteristics with each other

The 23 genetic characteristics gave 253 possible pairwise correlations. At the *p* < 0.05 level 12.7 would be expected to be associated by chance and 14 were observed to be associated at this level (O:E ratio = 1.1, *p* = 0.66). At the *p* < 0.01 level there were four observed associations compared to 2.53 expected, O:E = 1.6, *p* = 0.33.

### Associations of Genetic Characteristics with Nongenetic Characteristics

When we examined the association of each individual SNP with all 96 nongenetic factors, the observed pairwise correlations were similar to expected, with the exception of four variants at the α = 0.05 level and two variants at α = 0.01 (Tables 1 and 2). *APO_AV* was associated with 11 nongenetic characteristics at α = 0.05 (triglyceride levels, high-density lipoprotein cholesterol, fasting insulin, vitamin C, vitamin E, bilirubin levels, waist:hip ratio, age of the participant's mother when she died, area level deprivation, age at leaving full-time education, and outdoor ambient temperature in the month and location of birth of the participant), giving an O:E ratio of 2.3, *p* for difference O:E = 0.009. The number of significant associations of this variant with nongenetic characteristics at α = 0.01 was no greater than expected. The hepatic-lipase genetic variant was associated with ten nongenetic characteristics at α = 0.05 (triglyceride levels, high-density lipoprotein cholesterol, vitamin E, monocytes, phosphate levels, clotting factor VII, claudication, frequency of consumption of cheese, number of medications, and number of major diseases) giving an O:E of 2.1, *p* for difference O:E = 0.02. This variant was also associated with four of these nongenetic characteristics at α = 0.01 (triglycerides, vitamin E, clotting factor VII, and claudication), giving an O:E ratio at this level of significance of 2.5, *p* for difference O:E = 0.02.

The variant in the lipoprotein lipase gene was associated with ten nongenetic characteristics at α = 0.05 (triglyceride levels, high-density lipoprotein cholesterol, fasting insulin, eosinophils, bilirubin levels, frequency of consumption of fish, age at leaving full-time education, claudication, number of falls, and number of operations), giving an O:E ratio of 2.1, *p* for difference O:E = 0.02. This variant was also associated with four of these characteristics at α = 0.01 (triglycerides, high-density lipoprotein cholesterol, eosinophils, and claudication); O:E ratio 2.5, *p* for difference O:E = 0.02. Finally, variants in *TNFA* were associated with 11 nongenetic characteristics at α = 0.05 (trunk length, haemoglobin, mean cell volume, platelets, bilirubin, calcium, phosphate, fibrinogen, plasma viscosity, age of participant's mother when she died, and EuroQuol quality of life score), giving an O:E of 2.3, *p* for difference O:E = 0.009, with the number of associations of this variant with nongenetic characteristics at α = 0.01 being no greater than expected.

At α = 0.0001, each variant would be expected to be significantly associated with none (*n* = 0.0096) of the nongenetic variants. However, variation in the *lactase* gene was associated with mean outdoor temperature and rainfall in the area and month of the participants birth; variation in *CETP* was associated with high-density lipoprotein cholesterol; and variants in *LPL* were associated with triglyceride levels. The remaining 20 variants were not associated with any of the nongenetic characteristics at *p* ≤ 0.0001 (unpublished data).

Considering all 23 SNPs and 96 nongenetic factors (2,208 pairwise correlations), the number of expected significant correlations by chance would be 110, 22.1, and 0.221 at α = 0.05, 0.01, and 0.0001, respectively. We observed values similar to this at α = 0.05 (*n* = 120; *p* for difference between observed and expected = 0.35) and α = 0.01 (*n* = 27; *p* for difference between observed and expected = 0.28), and higher than expected at α = 0.0001 (*n* = 4; *p* for difference between observed and expected = 0.00008).

## Discussion

Over 50% of the pairwise associations between baseline nongenetic characteristics in our study were statistically significant at the 0.05 level; an 11-fold increase from what would be expected, assuming these characteristics were independent. Similar findings were found for statistically significant associations at the 0.01 level (45-fold increase from expected) and the 0.0001 level (3,000-fold increase from expected). This illustrates the considerable difficulty of determining which associations are valid and potentially causal from a background of highly correlated factors, reflecting that behavioural, socioeconomic, and physiological characteristics tend to cluster. This tendency will mean that there will often be high levels of confounding when studying any single factor in relation to an outcome. Given the complexity of such confounding, even after formal statistical adjustment, a lack of data for some confounders, and measurement error in assessed confounders will leave considerable scope for residual confounding [4]. When epidemiological studies present adjusted associations as a reflection of the magnitude of a causal association, they are assuming that all possible confounding factors have been accurately measured and that their relationships with the outcome have been appropriately modelled. We think this is unlikely to be the case in most observational epidemiological studies [26].

Predictably, such confounded relationships will be particularly marked for highly socially and culturally patterned risk factors, such as dietary intake. This high degree of confounding might underlie the poor concordance of observational epidemiological studies that identified dietary factors (such as beta carotene, vitamin E, and vitamin C intake) as protective against cardiovascular disease and cancer, with the findings of randomized controlled trials of these dietary factors [1,27]. Indeed, with 45% of the pairwise associations of nongenetic characteristics being “statistically significant” at the *p* < 0.01 level in our study, and our study being unexceptional with regard to the levels of confounding that will be found in observational investigations, it is clear that the large majority of associations that exist in observational databases will not reach publication. We suggest that those that do achieve publication will reflect apparent biological plausibility (a weak causal criterion [28]) and the interests of investigators. Examples exist of investigators reporting provisional analyses in abstracts—such as antioxidant vitamin intake being apparently protective against future cardiovascular events in women with clinical evidence of cardiovascular disease [29]—but not going on to full publication of these findings, perhaps because randomized controlled trials appeared soon after the presentation of the abstracts [30] that rendered their findings as being unlikely to reflect causal relationships. Conversely, it is likely that the large majority of null findings will not achieve publication, unless they contradict high-profile prior findings, as has been demonstrated in molecular genetic research [31].

The magnitudes of most of the significant correlations between nongenetic characteristics were small (see Figure 1), with a median value at *p* ≤ 0.01 and *p* ≤ 0.05 of 0.08, and it might be considered that such weak associations are unlikely to be important sources of confounding. However, so many associated nongenetic variables, even with weak correlations, can present a very important potential for residual confounding. For example, we have previously demonstrated how 15 socioeconomic and behavioural risk factors, each with weak but statistically independent (at *p* ≤ 0.05) associations with both vitamin C levels and coronary heart disease (CHD), could together account for an apparent strong protective effect (odds ratio = 0.60 comparing top to bottom quarter of vitamin C distribution) of vitamin C on CHD [32].

The independence of genetic and environmental factors is of importance in other domains of genetic epidemiology, in addition to that of mendelian randomization. First, case-only studies necessarily assume the independence of genetic and environmental factors in their basic rationale [33,34]. Second, statistical methods for analysing case-control studies in genetic epidemiology can enhance precision by assuming the independence of genetic and environmental factors, as demonstrated by several authors [35–37]. Such approaches have been applied to the analysis of empirical datasets [38]. Conversely, it is commonplace to see statistical adjustment for environmental factors applied to associations between genetic variants and outcomes. This adjustment is probably unnecessary, given the independence of the genetic variants and the environmental factors, and it also provides opportunity for data-derived selection of the adjusted model that provides the strongest evidence for an association with the genetic variant in question. In some cases, indeed, only the adjusted analyses are presented. We suggest that routine adjustment of genetic associations with phenotypic outcomes for potential nongenetic confounding factors is unnecessary and can be misleading.

Three of the authors decided a priori which baseline characteristics were likely to be biologically closely related to each other or likely to be measuring the same underlying characteristic and did not include such variables in the overall correlations. Other investigators might have come up with somewhat different grouping of variables. However, the very high proportion of statistically significant associations at all three levels of significance and the similar findings with sensitivity analyses using different nongenetic characteristics (e.g., total cholesterol instead of triglycerides, high-density lipoprotein cholesterol and low-density lipoprotein cholesterol) suggest that our findings are likely to be replicated even with different opinions about which baseline nongenetic variables should be included in the analyses (provided this selection of nongenetic variables was done a priori within any given dataset).We also deliberately chose only one genetic variant when we had typed several within a gene; this selection ensured there is no association caused by linkage disequilibrium due to close physical proximity of variants. It is possible that pleiotropy or population stratification could generate associations between genetic variants and nongenetic factors, but we do not see strong evidence of this in our study population of United Kingdom (UK) women, very largely of white European origin.

The genetic polymorphisms that we investigated were those that had been assayed in this cohort study. The variants that we have typed to date are those that we (or study collaborators) wish to use in mendelian randomization studies or to replicate previous association studies. Thus, these variants have all been selected on the grounds that there was some evidence that they relate to biological differences between individuals for phenotypes or disease outcomes that we have assessed in this cohort. Therefore, they are a group of variants that will tend to be related to phenotypic differences. Our variants include, for example, the C→T677 *MTHFR* variant and the SNP that marks the lactase persistence trait, two well-known and widely studied variants with clear biological correlates. The number of associations found with phenotypic variables should, therefore, be higher for our SNPs than for a group of SNPs selected without reference to known function. Four of the chosen variants (lying at the *APO_AV*, *HL*, *LPL*, and *TNFA* loci) were associated with more phenotypes than expected at either the 0.05 or 0.01 significance level. It is possible that these variants are involved in such a wide range of biological processes that the observations are causal. However, these “positive” findings, particularly those at the 0.05 level, may well simply represent the play of chance and be nonreplicable in future studies. In support of our general hypothesis that in mendelian randomization studies, genetic variants are seldom confounded by phenotypic factors [10], overall we found no more associations with phenotypes than would be expected by chance at the 0.05 or 0.01 level.

At a more realistic *p*-value threshold for genetic association studies (*p* ≤ 0.0001), only four (0.18%) out of 2,208 associations of 23 genetic variants with 96 nongenetic variants were statistically significant. Although this is greater than the number (0.22) expected by chance, the proportion of statistically significant associations of genotype with nongenetic characteristics is considerably smaller than the proportion of significant associations between nongenetic characteristics (0.18% versus 30%) at this level of significance. It is difficult to believe that all or a substantial proportion of the 1,378 statistically significant associations (at *p* ≤ 0.0001) between two nongenetic characteristics are truly causal, whereas the four associations of genetic variants with nongenetic factor associations at this level of significance may well be real. The association of variants in *lactase* with mean outdoor temperature and rainfall for the area and month of birth of the participant is likely to reflect the established population stratification for this variant [39,40] Since the allele frequency of this variant is known to vary by ancestral geography, we would take this into account in any mendelian randomization studies of this variant. The other two associations—*CETP* with high-density lipoprotein cholesterol [41–43]; and *LPL* with triglycerides [44]—reflect the biological actions of these genes.

Our findings provide reassuring evidence that utilising genetic variants in mendelian randomization studies is generally a legitimate strategy. Furthermore, statistical methods that assume independence of genetic and environmental factors are also legitimate in many circumstances [33–38]. Our findings are concordant with the demonstration that a large number of genetic variants were unrelated to participation or nonparticipation in a series of case-control studies [45]; with occasional reports of gene–environment independence that have focused on a limited number of variants and environmental factors [46]; with the very similar distribution of allelic frequencies among blood donors and a representative population sample in the UK [47] and with a detailed review of gene–environment correlations in behavioural genetics [48]. We have demonstrated a fundamental difference in the degree of confounding of genetic variants and other variables. This difference can be exploited by using genetic variants as exposure indicators to study the effects on common diseases of modifiable risk factors that are too heavily confounded to be studied robustly through conventional observational epidemiological approaches [10].

## Supporting Information

Table S1 Phenotypes Included in Primary Analyses (38 KB DOC)

Table S2 Constituent Phenotypes Used in the Main Analysis (27 KB DOC)

Table S3 Genotypes Used in the Main Analyses (46 KB DOC)

Text S1 Phenotypes Included in Main Analyses (35 KB DOC)

## Abbreviations

O:E: observed to expected
SNP: single nucleotide polymorphism
