# Supplementary material for: Clustered Environments and Randomized Genes: A Fundamental Distinction between Conventional and Genetic Epidemiology
Source: PLoS Med. 2007 Dec 11;4(12):e352. doi: 10.1371/journal.pmed.0040352 (PMC2121108; doi:10.1371/journal.pmed.0040352)
Supplement: Supplementary file 1 [file pmed.0040352.sd001.doc]

Supporting Text S1: Phenotypes included in main analyses

1. Area deprivation score (continuous)
2. Leg length (continuous) ^1^
3. Trunk length (continuous) ^1^
4. Body mass index (continuous) ^2^
5. Waist to hip ratio (continuous)
6. Systolic blood pressure (continuous) ^2^
7. Pulse (continuous)
8. ECG ischaemia categories (ordered categorical: none, possible, probable, definite)^2^
9. Forced expiratory volume in 1 second (continuous) ^2^
10. Haemoglobin (continuous) ^2^
11. Mean cell volume (continuous)
12. Platelets (continuous)
13. Neutrophils (continuous - logged) ^1^
14. Lymphocytes (continuous) ^1^
15. Monocytes (continuous) ^1^
16. Eosinophils (continuous - logged) ^1^
17. Basophils (continuous - logged) ^1^
18. Urea (continuous - logged) ^2^
19. Sodium (continuous) ^2^
20. Urate (continuous)
21. Magnesium (continuous)
22. Calcium (continuous)
23. Phosphate (continuous)
24. Albumin (continuous) ^2^
25. Bilirubin total (ordered categorical - fifths)
26. Aspartate Transaminase (continuous) ^2^
27. High density lipoprotein cholesterol (continuous) ^1^
28. Low density lipoprotein cholesterol (continuous) ^1^
29. Triglycerides (continuous – logged) ^1^
30. Vitamin C (continuous)
31. Vitamin E (ordered categorical - fifths)
32. Fasting insulin (continuous - logged)
33. Fasting glucose (continuous – logged) ^2^
34. Fibrinogen (continuous)
35. Tissue Plasminogen activator (continuous)
36. Von Willibrands (continuous)
37. Clotting factor VII (continuous)
38. Clotting factor VIII (continuous)
39. Clotting factor IX (continuous)
40. D-dimer (ordered categorical – fifths)
41. C-reactive protein (continuous - logged)
42. IL6 (ordered categorical - fifths)
43. Plasma viscosity (continuous)
44. Euroquol quality of life score (ordered categorical – fifths)
45. Type of bread eaten (would make binary – wholemeal yes/no)
46. Type of spread used (ordered categorical based on fat content: none, low-fat only, full-fat butter or margarine)
47. Type of milk used (ordered categorical based on fat content: none, skimmed only, semi-skimmed only, full-fat)
48. Frequency consumption fresh fruit (ordered categorical: >=1 per day; most days; 1-2 days per week; < once per week)
49. Frequency consumption fresh vegetables (ordered categorical: >=1 per day; most days; 1-2 days per week; < once per week)
50. Frequency consumption salad (ordered categorical: >=1 per day; most days; 1-2 days per week; < once per week)
51. Frequency fish (ordered categorical: >=1 per day; most days; 1-2 days per week; < once per week)
52. Frequency poultry (ordered categorical: >=1 per day; most days; 1-2 days per week; < once per week)
53. Frequency red meat (ordered categorical: >=1 per day; most days; 1-2 days per week; < once per week)
54. Frequency processed meat (ordered categorical: >=1 per day; most days; 1-2 days per week; < once per week)
55. Frequency cereals (ordered categorical: >=1 per day; most days; 1-2 days per week; < once per week)
56. Frequency nuts (ordered categorical: >=1 per day or most days; 1-2 days per week; < once per week)
57. Frequency cheese (ordered categorical: >=1 per day; most days; 1-2 days per week; < once per week)
58. Hours per week of heavy housework (ordered categorical – fifths)
59. Hours per week of heavy gardening (ordered categorical – fifths)
60. Hours per week of jogging or vigorous exercise (ordered categorical – fifths)
61. Hours per week of do-it-yourself work (DIY) (ordered categorical – fifths)
62. Walking speed (ordered categorical – slow, steady/average, brisk, fast (at least 4mph))
63. Childhood social class (ordered categorical 6 categories I-V) ^2^
64. Age at leaving full-time education (ordered categorical: <=14, 15-16, 17-18, >=19 years)
65. Lived in a house with no bathroom as a child (binary) ^2^
66. Shared bedroom as child (binary)
67. Housing tenure as a adult (ordered categorical: own home, private rental, public (social housing))
68. Access to a car as an adult ^2^
69. Income arrangements for retirement (binary state pension only versus state + other pension)
70. Claudication score (ordered categorical: none, possible, probably, definite claudication)
71. Smoking (ordinal categories – 3: never, past, current)
72. Weight at age 21 (continuous) ^2^
73. Weight change in last four years (ordered categorical: <= –28 or less, -27 to 14, -13 to –1, 0, 1-13, 14 to 27, >=28 lbs)
74. Alcohol intake (ordered categorical: teetotal & quarters of distribution of drinks per week for drinkers)
75. Marital status (binary: currently married versus single, widowed, divorced/separated, other)
76. Age menarche (continuous)
77. Age menopause (continuous)
78. Number pregnancies (ordered categorical: 0,1,2,3,4,>=5)
79. Age of participants father when he died (continuous)
80. Age of participants mother when she died (continuous)
81. Offspring birth weight (continuous)
82. Own birth weight (continuous)
83. Use of hormone replacement (ordered categorical: never, past, current)
84. Health at present (ordered categorical: poor, fair, good, excellent)
85. Number of falls in last 12 months (ordered categorical: 0, 1, 2, >=3)
86. Limitations of activities of daily living score (ordered categorical: 0, 1, 2, 3, >=4)
87. Social contact score (ordered categorical: fifths)
88. Number of siblings (ordered categorical: 0, 1, 2, 3, 4, 5, >=6)
89. Height of husband (continuous)
90. Husband’s smoking status (ordered categorical – never, past, current)
91. Number medications (ordered categorical: 0, 1, 2, 3, 4, 5, >=6)
92. Number of major illnesses (ordered categorical: 0, 1, 2, >=3)
93. Number of surgical operations (ordered categorical: 0, 1, 2, 3, >=4)
94. Mean temperature in month of birth (continuous)
95. Mean rainfall in month of birth (continuous)
96. Nurse estimation of life expectancy (continuous) ^2^

^1^ Phenotypes that are constituents of an overall phenotype that was not included in the main analyses. In a series of sensitivity analyses all of these variables were removed and replaced by the overall variable (See Table 1 below).

^2^ Characteristics that are measuring the underlying phenotype. In the first column the randomly selected characteristic that was used in the primary analysis is given. In a series of sensitivity analyses these variables were removed and replaced by one of the equivalent variables from the second column (See Table 2 below).
