# Supplementary material for: Clustered Environments and Randomized Genes: A Fundamental Distinction between Conventional and Genetic Epidemiology
Source: PLoS Med. 2007 Dec 11;4(12):e352. doi: 10.1371/journal.pmed.0040352 (PMC2121108; doi:10.1371/journal.pmed.0040352)
Supplement: Supplementary file 2 [file pmed.0040352.st001.doc]

Supporting Table S1: Phenotypes included in primary analyses

| Phenotype used in primary analysis | **Phenotypes considered a priori to be linked to the phenotype included in the primary analysis** |
| --- | --- |
| BMI | Current weight  Current dress size |
| Systolic BP | Diastolic BP |
| ECG ischaemia | Rose Angina |
| FEV_1_ | Peak Expiratory Flow  Forced Vital Capacity  Forced Expiratory Flow rate during end of expiration  Forced Expiratory Flow rate during middle of expiration |
| Haemoglobin | Mean Cell Haemoglobin  Red Cell count  Haematocrit  Mean Packed volume |
| Urea | Creatinine |
| Sodium | Potassium |
| Albumin | Total Protein |
| Aspartate Transaminase (AST) | Alkaline phosphatase (ALP)  Alkaline Aminotransferase (ALT)  Gamma-glytamyl transpeptidase (GGT) |
| Fasting Glucose | Glycated Haemoglobin |
| Childhood social class | Adulthood social class |
| Lived in house with bathroom as a child | Lived in house with hot water as a child |
| Weight at 21 | Dress size at 21 |
| Access to a car as a child | Access to a car as an adult |
| Nurse estimated life expectancy | Nurse estimated current age |
