# Supplementary material for: Clustered Environments and Randomized Genes: A Fundamental Distinction between Conventional and Genetic Epidemiology
Source: PLoS Med. 2007 Dec 11;4(12):e352. doi: 10.1371/journal.pmed.0040352 (PMC2121108; doi:10.1371/journal.pmed.0040352)
Supplement: Supplementary file 3 [file pmed.0040352.st002.doc]

# Supporting table S2: Constituent phenotypes used in the main analysis

| **Constituent phenotypes included in main analyses presented here** | **Overall phenotype that was included in sensitivity analyses instead of all constituent phenotypes** |
| --- | --- |
| Leg length  Trunk length | Standing height |
| Neutrophils  Lymphocytes  Monocytes  Eosinophils  Basophils | Total white cell count |
| High density lipoprotein cholesterol  Low density lipoprotein cholesterol  Triglycerides | Total cholesterol |
