# Supplementary material for: Clustered Environments and Randomized Genes: A Fundamental Distinction between Conventional and Genetic Epidemiology
Source: PLoS Med. 2007 Dec 11;4(12):e352. doi: 10.1371/journal.pmed.0040352 (PMC2121108; doi:10.1371/journal.pmed.0040352)
Supplement: Supplementary file 4 [file pmed.0040352.st003.doc]

# Supporting table S3: Genotypes used in the main analyses

All of these were treated as a score from 0 to 2 (0=homozygous for common allele, 1=heterozygous, 2=homozygous for rare allele)

Genotypes in the second column were used with the main genotype included in the analyses to tag a haplotypes block.

| **SNPs included in main analyses** | **Other SNPs genotyped to tag a haplotype block** |
| --- | --- |
| LAC1 (rs4988235) | LAC2 (rs182549) |
| CETP (rs708272) | None |
| APO_AV (rs3135506) | APO_AV (rs662799)  APO_AV (rs1021)  APO_AV (rs1022) |
| HL (rs1800588) | None |
| LPL (rs328) | LPL (rs1018) |
| TNF-α (rs1800629) | TNF-α (rs361525) |
| LTA (rs1041981) | LTA (rs909253)  LTA (rs2239704)  LTA (rs3093546) |
| LGAL (rs7291467) | None |
| ALOX5AP (rs1004) | ALOX5AP (rs1005)  ALOX5AP (rs1006) |
| GPX4 (rs1007) | None |
| IL-6 (rs1800795) | IL-6 (rs1016) |
| PTGS2 (rs20417) | None |
| ESR1 (rs2234693) | ESR1 (rs9340799) |
| MTHFR (rs1801133) | None |
| MEF2A (rs3730059) | MEF2A (rs325400) |

| **SNPs included in main analyses** | **Other SNPs genotyped to tag a haplotype block** |
| --- | --- |
| ADIPOQ (rs1501299) | ADIPOQ (rs2241766)  ADIPOQ (rs822395)  ADIPOQ (rs1008) |
| PPPIR (rs854541) | None |
| GCK (rs1799884) | GCK (rs3757840)  GCK (rs1019)  GCK (rs1020) |
| ACE (rs4343) | None |
| Elastin (rs2071307) | None |
| PON1 (rs662) | None |
| GHRLL (rs696217) | GHRLL (rs605353)  GHRLL (rs4684677) |
| PTC (rs713598) | PTC (rs1726866) |
